# Plasma N-acetylputrescine, cadaverine and 1,3-diaminopropane: potential biomarkers of lung cancer used to evaluate the efficacy of anticancer drugs

**DOI:** 10.18632/oncotarget.19304

**Published:** 2017-07-17

**Authors:** Ran Liu, Pei Li, Cathy Wenchuan Bi, Ran Ma, Yidi Yin, Kaishun Bi, Qing Li

**Affiliations:** ^1^ School of Pharmacy, Shenyang Pharmaceutical University, Shenyang, China; ^2^ Division of Life Science and Center for Chinese Medicine, The Hong Kong University of Science and Technology, Hong Kong, China; ^3^ School of Basic Medical Sciences, Liaoning University of Traditional Chinese Medicine, Shenyang, China

**Keywords:** polyamine, SCCL, cancer and medication biomarkers, targeted metabolomics

## Abstract

Polyamines have been widely investigated as potential biomarkers for various types of cancers, including lung cancer, which is one of the most common causes of death from cancer worldwide. This study was carried out to evaluate the value of polyamines that serve as early diagnostic and cancer progression markers as well as drug evaluation for lung cancer (squamous cell carcinoma of lung, SCCL). SCCL was induced in Wistar rats by intratracheal instillation of 3-methylcholanthrene and treated with three different anti-cancer drugs, Aidi injections, fluorouracil, and a combination of them. After carcinogenesis for 28, 70 and 98 days and therapy for 28 and 56 days, the polyamine levels in plasma of SCCL, healthy and treated rats were determined using a UHPLC-MS/MS assay base on the means of targeted metabolomics. Results showed that increased N-acetylputrescine, cadaverine and 1,3-diaminopropane levels were associated with progression of SCCL. The levels of cadaverine and 1,3-diaminopropane returned to normal after administration of the three different kinds of anticancer drug. In addition, the suitability of using N-acetylputrescine, cadaverine and 1,3-diaminopropane as biomarkers was confirmed by PLS-DA and ROC analysis. It can provide an innovative and effective way for the clinical diagnosis, prevention and treatment of lung cancer, and stimulate a theoretical basis for the design and development of new anticancer drugs. At the same time, this increased the clinical options for polyamines as cancer biomarkers.

## INTRODUCTION

Lung cancer has been the most common form of cancer worldwide for several decades, and it is also the most common cause of death from cancer [1–3]. Detailed pathogenesis, effective early detection and the use of suitable drugs help in the effective therapy of lung cancer. Among these, the early recognition of lung cancer is crucial, especially in screening the high-risk populations, as more aggressive treatment may improve clinical outcomes. Also, accurate diagnosis is vital for the most suitable treatment of individual patients with lung cancer. Thus, there is an urgent need to identify sensitive and specific biomarkers for early diagnosis and the therapeutic targets for investigating the pathogenetic mechanism of lung cancer. At present, the two protein biomarkers, CYFRA21-1 and SCCAg, are the most widely used forthe early diagosis of lung cancer [4–6]. However, a single biosignature is not enough to distinguish between healthy and diseased individuals. Thus, a combination of biosignatures, such as antigens, carbohydrates, enzymes, steroids and small molecule cancer markers, could improve the chances of an early diagnosis [7–10].

Metabolomics is now an established methodology and has wide ranging applications in many fields. It aims to profile all low-molecular weight metabolites that are present in biological samples to increase understanding of the effect of a particular stimulus, from the disease or drug, which affects metabolic pathways [11]. Targeted and untargeted applications are used for the identification of etiological pathways and the discovery of biomarkers for the prediction, diagnosis, and prognosis of major chronic diseases [12]. Through targeted metabolomics, the identified pathways or biomarkers could be confirmed by quantification of a limited number of metabolites in samples which leads to improved limits of detection and quantification compared with the use of open profiling approaches to confirm the biomarkers [13–15].

Among the different identified pathways in living organisms, polyamines are essential for the cell growth and differentiation of normal and neoplastic tissues. Polyamines, including putrescine, cadaverine, spermidine, spermine, agmatine, N-acetylputrescine, N-acetylspermine, N-acetylspermidine and 1, 3-diaminopropane, are metabolized by amino acid such as L-ornithine, lysine and L-arginine. An increasing number of reports in the literature have now identified polyamine catabolism is a contributory factor in the development of a variety of cancers [16–20]. Regarding research into the relationship between polyamine metabolic profiling and lung cancer, we recently published papers on the level of polyamine metabolome in human subjects who suffered from lung and liver cancer [21–23]. Our results demonstrated the significance of polyamine metabolism in lung cancer. Because of the difficulties from clinical sample collection, the role of early diagnosis and therapeutic evaluation of polyamines in lung cancer had not been carried out. However, it encouraged us to undertake this study to determine a possible interrelation between lung carcinoma cell proliferation and polyamine metabolism in the early stages of lung cancer. Furthermore, we also examined potential therapeutic applications by investigating potential pharmaceutical interventions in polyamine metabolism.

Currently, with advantages of multi-target and multi-coordinated system, Chinese medicine is being widely used in cancer treatment, and positive effects have been achieved. However, problems including incomplete functional mechanisms, have affected the application of compound traditional Chinese medicines (CTCM). So, it is important to carry out CTCM anticancer pharmacodynamic evaluation on the basis of a polyamine metabolism response spectrum, which will provide new information about the correlation between biological endogenous substances and CTCM pharmacodynamics.

In this report we described how we investigated the impact of lung cancer on polyamine metabolism by examining the plasma from 3-methylcholanthrene (MCA) induced SCCL rats. Then, we evaluated the altered polyamine metabolism in the presence of an anti-cancer medicine, a complex Chinese medicine, a commonly used chemotherapeutic treatment and a combination of them. By targeted metabolomics analysis, simultaneous determination of polyamine metabolic profiles to identify specific biomarkers for the early diagnosis of SCCL and sensitive biomarkers for anti-cancer monitoring had been developed innovatively as well as constructively. The results obtained could provide an effective way for the clinical diagnosis, prevention and treatment of lung cancer, and provide a theoretical basis for the design and development of new anticancer drugs. Furthermore, this increased the clinical options for polyamines as biomarkers of cancer.

## RESULTS

### Histopathology study of SCCL rats and treated rats

The rats with SCCL induced by MCA solution were similar to human SCCL [26, 27]. MCA solution was prepared in Iodinated oil Injection at a temperature of 70–80°C and 10% (V/V) diethylnitrosamine was added to promote cancer development. Then, 0.1 ml MCA solution (containing 10 mg MCA and 0.01 ml DEN) was instilled once intratracheally into the left inferior pulmonary lobe of the rats.

**Figure 1:**
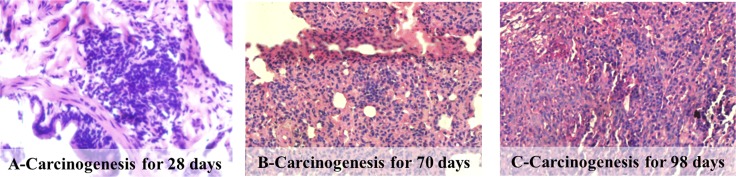
Histopathological photomicrographs of pathological lung sections obtained after intraperitoneal injection of the mixture of MCA and DEN solutions (haematoxylin and eosin (HE), ×40)

General observation and histopathologic examinations were used for the evaluation of the SCCL model. A significant tumor was found on the left inferior pulmonary lobe of the SCCL rats, identified by general observation, but no primary or metastatic tumor was found on the other organs while there were no differences in the incidence of any lesions between the three treatment and normal groups. Histopathologic examination identified marked multifocal nests after carcinogenesis for 28 days (Figure 1A). With the process of carcinogenesis, the cancer cells increased in volume and number, with the development of cystic lesions (Figure 1B) and infiltration into the surrounding lung tissue (Figure 1C), which proved that the SCCL model had been successfully established.

**Figure 2:**
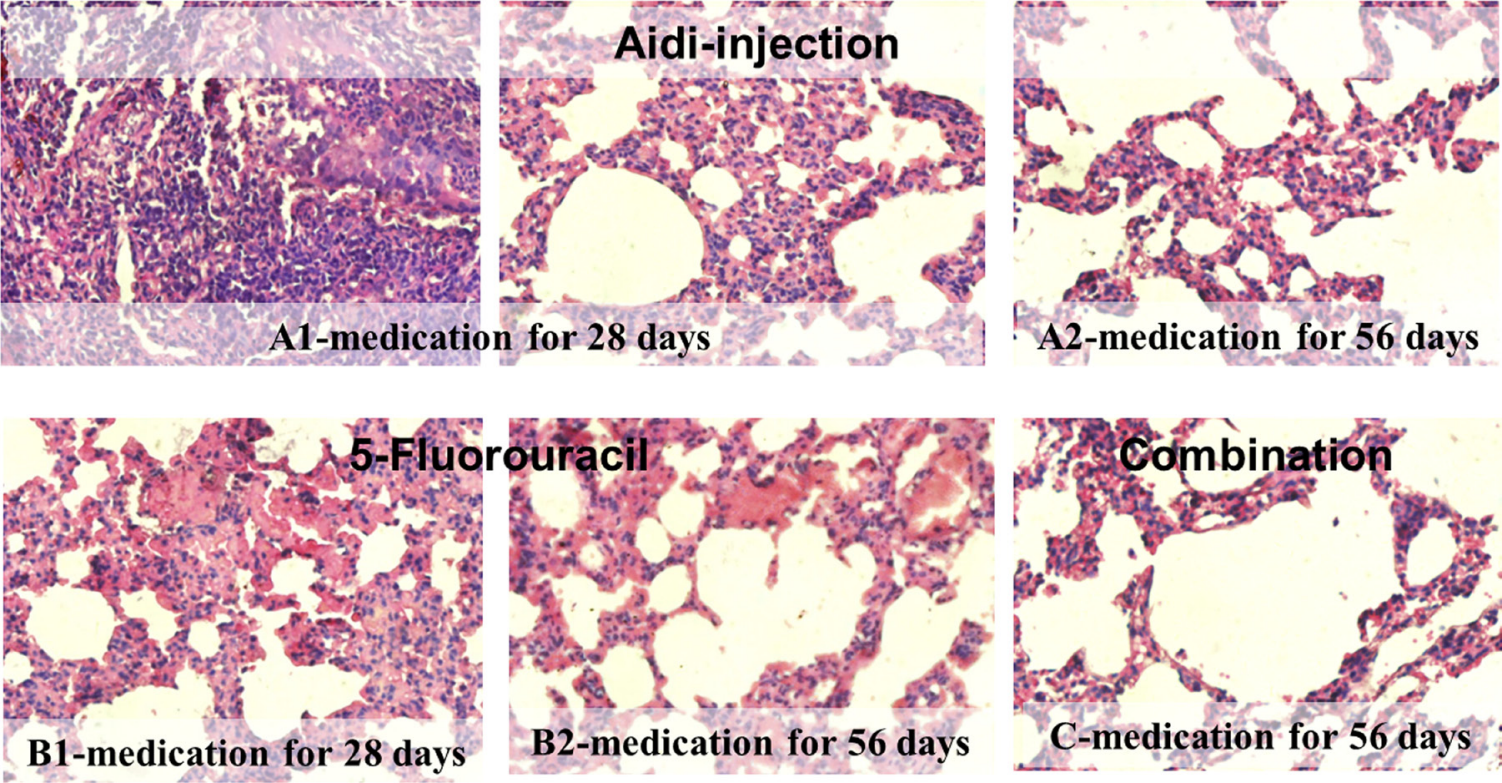
Histopathological photomicrographs of lung cancer rats after therapeutic treatment (HE, ×40) (**A**) Aidi-injection; (**B**) 5-Fluorouracil; (**C**) Combination treatment.

As shown in Figure 2, the left inferior pulmonary lobe of the treated rats were also examined by obtaining pathological sections. On the 70th day, the MCA rats were given an Aidi injection for 28 days, and it could be seen that the cancer cells had been taken up by inflammatory cells as well as infiltrated many lymphocytes and multinuclear giant cells (Figure 2A1). After treatment for 56 days, alveolar cells were clearly seen and pulmonary cells tended to return to normal (Figure 2A2), which indicated the effect on self-recovery and immunity was increased by Aidi injection. When the MCA infected rat were treated with 5-fluorouracil for 28 days, necrotic cancerous cells and non-inflammatory cells were observed (Figure 2B1). While, after treatment for 56 days, the pulmonary cells tend to return normal (Figure 2B2). However, importantly, the rats given combinated treatments exhibited self-recovery and their immunity were enhanced. It seems that the secondary reaction of the chemotherapeutic, 5-fluorouracil, might be reduced by the anti-cancer Chinese medicine. So, normal alveolar cells were seen in the rats given combined treatment during their period of medication (Figure 2C).

### Levels of CYFRA21-1 and SCCAg in the plasma of SCCL and treated rats

Many studies have reported that CYFRA21-1(cytokeratinfragment 21-1) and SCCAg (squamous cell carcinoma associated antigen) can be used as specific biomarkers for the early diagnosis of SCCL[4–6]. However, there are few reports about the application to the screening of the therapeutic effects of anti-cancer drugs. So, the changes in the expression of CYFRA21-1 and SCCAg were studied during the stage of SCCL onset, development and treatment as well as confirmation of the biomarker role of polyamines.

The levels of CYFRA21-1 and SCCAg in rat plasma were measured by enzyme linked immunosorbent assay (ELISA) and the results are shown in Table 1. Continuously increased levels were found in SCCL rats compared with normal rats (*P <* 0.01), even during the initial stage of SCCL. Reduced levels of CYFRA21-1 and SCCAg were found during anti-cancer drug administration. Comparison of rats given Aidi-injection, 5-fluorouracil and combined treatment showed that there was no significant difference in CYFRA21-1 and SCCAg in the early stage of therapy. However, as the therapy continued, the levels decreased in the rats given combination treatment. These results showed the feasibility of screening the anti-cancer drug effect when CYFRA21-1 and SCCAg were used as markers, while the Aidi injection and the combination of Aidi injection and 5-fluorouracil had an effect on the SCCL.

**Table 1:** Level of CYFRA21-1 and SCCAg in the different groups ( *x* ± s) *n* = 16

Group	CYFRA21 (ng/ml)	SCCAg (ng/ml)
Normal Rats	0.096 ± 0.011	0.865 ± 0.092
SCCL Rats	0.174 ± 0.004*	0.180 ± 0.062*
Aidi-injection treated rats	0.102 ± 0.006	0.937 ± 0.096
5-fluorouracil treated rats	0.097 ± 0.009	0.866 ± 0.034
combination treated rats	0.098 ± 0.020	0.900 ± 0.031

*p < 0.05, compared with normal rats.

### Polyamine metabolic profile in SCCL and treated rats

The levels of 14 polyamine metabolomes in each plasma sample were determined from calibration curves (Supplementary Table 3 listed the detailed results of the method validation). Table 2 summarizes the concentrations (mean ± SD) of the fourteen plasma polyamine metabolomes in SCCL rats and normal rats. On the 28th day after carcinogenesis, compared with normal rats, most of the polyamine levels in the rats with cancer were similar to those of the normal rats except for an increased level of putrescine and N-acetylputrescine as well as reduced S-adenosyl-L-methionine (*p* < 0.05). This suggested that when the lung cell carcinoma appeared, the polyamine metabolism, especially the pathway related to putrescine, was increased which was why we found increased levels of putrescine in body fluids. Similarly, on the 70th day after carcinogenesis, the polyamine levels were significantly increased for cadaverine, N-acetylputrescine, 1,3-diaminopropane and L-ornithine. It seems that the consumption of putrescine, especially the pathway from L-ornithine to N-acetylputrescine, was disturbed during the SCCL period. On the 98th day after carcinogenesis, compared with normal rats, the polyamine levels in the SCCL rats were increased for cadaverine, N-acetylputrescine and 1,3-diaminopropane. It has been shown that the increased polyamine metabolism is a typical feature of lung caner malignancy, and the catabolism or transmission of N-acetylputrescine seems to be interfered obviously. In summary, N-acetylputrescine can be used as a potential biomarker of SCCL.

**Table 2:** Amounts of polyamine metabolomes in plasma (ng/mL) from Normal Rats (*n* = 8) and SCCL Rats (*n* = 8) from the 28th day to the 98th day during the experiment

	28th day		70th day		98th day	
	Normal Rats	SCCL Rats	Normal Rats	SCCL Rats	Normal Rats	SCCL Rats
1,3-diaminopropane	114.7 ± 22.1	135.1 ± 9.7	90.9 ± 4.7	928 ± 458*	381.2 ± 110.9	1430 ± 163*
putrescine	3997 ± 1083	4345 ± 1097	3521 ± 560	5288 ± 1812	3708 ± 1054	3194 ± 1751
cadaverine	383.7 ± 106.0	757.6 ± 45.1*	258.3 ± 119.5	990 ± 300*	1300 ± 503	4984 ± 1371*
spermine	6.623 × 10^4^ ± 0.534 × 10^4^	4.830 × 10^4^ ± 1.816 × 10^4^	2.204 × 10^4^ ± 0.475 × 10^4^	1.950 × 10^4^ ± 1.627 × 10^4^	1.497 × 10^4^ ± 0.708 × 10^4^	1.590 × 10^4^ ± 0.564 × 10^4^
spermidine	1.391 × 10^4^ ± 0.118 × 10^4^	9.78 × 10^3^ ± 1.94 × 10^4^	7088 ± 4494	6373 ± 4061	6925 ± 2124	7804 ± 2546
N-acetylputrescine	2.51 ± 1.23	5.11 ± 0.11*	3.82 ± 0.58	6.95 ± 1.48*	3.06 ± 1.02	6.19 ± 1.03*
N-acetylspermine	2.39 ± 1.06	2.01 ± 0.29	2.25 ± 0.25	3.34 ± 2.13	1.40 ± 0.28	1.31 ± 0.32
N-acetylspermidine	2.29 ± 0.80	1.22 ± 0.33	2.07 ± 0.93	2.04 ± 1.29	1.04 ± 0.36	1.93 ± 0.84
γ-aminobutyric acid	1996 ± 521	2289 ± 105	2056 ± 593	4154 ± 5452	1725 ± 761	1415 ± 673
agmatine	52.90 ± 6.75	58.33 ± 3.84	48.46 ± 7.55	75.93 ± 45.61	77.34 ± 83.42	43.90 ± 12.91
L-arginine	2.001 × 10^4^ ± 0.279 × 10^4^	1.468 × 10^4^ ± 0.244 × 10^4^	1.928 × 10^4^ ± 0.263 × 10^4^	1.623 × 10^4^ ± 1.032 × 10^4^	1.659 × 10^4^ ± 0.535 × 10^4^	1.750 × 10^4^ ± 0.225 × 10^4^
lysine	3.066 × 10^4^ ± 0.833 × 10^4^	3.377 × 10^4^ ± 0.376 × 10^4^	2.441 × 10^4^ ± 0.625 × 10^4^	3.386 × 10^4^ ± 0.716 × 10^4^	2.169 × 10^4^ ± 0.547 × 10^4^	2.467 × 10^4^ ± 1.064 × 10^4^
L-ornithine	1.874 × 10^4^ ± 0.984 × 10^4^	5.236 × 10^4^ ± 2.258 × 10^4^	1.376 × 10^4^ ± 0.369 × 10^4^	6.198 × 10^4^ ± 2.650 × 10^4^*	6.505 × 10^4^ ± 4.269 × 10^4^	4.061 × 10^4^ ± 2.977 × 10^4^
S-adenosyl-L-methionine	357.6 ± 19.7	282.0 ± 6.0*	160.8 ± 11.3	246.7 ± 87.3	116.2 ± 45.8	152.8 ± 60.6

(mean ± SD). *p < 0.05, compared with normal rats.

As indicated in Table 3, the concentrations (mean ± SD) of the fourteen plasma polyamine metabolomes in treated rats differed significantly according to the treatments used. On the 70th day after carcinogenesis, when the rats has been given anti-cancer drugs for 28 days, the levels of cadaverine and 1,3-diaminopropane were significantly reduced in all the three drug treated rats, compared with SCCL rats. In addition, there were increased levels of N-acetylputrescine and spermine in 5-fluorouracil treated rats, and a decreased level of lysine as well as an increased level of spermine in the rats given combination treatment. Then, after being given anticarcinogen treatment for about 56 days, slight alterations in plasma polyamine levels were observed in the rats given the three different reatments. Compared with SCCL rats, the levels of cadaverine and 1,3-diaminopropane were sequentially reduced in all the treated rats. Meanwhile, in the 5-fluorouracil treated rats, a reduced level of N-acetylputrescine as well as increased levels of N-acetylspermine and L-ornithine were observed. The changes in polyamine levels differed between the rats given Aidi injections and 5-fluorouracil injections. This might be because the different anti-cancer drugs had different mechanisms of action involving cancer therapy.

**Table 3:** Amounts of polyamine metabolomes in plasma (ng/mL) from Aidi injection treated rats (*n* = 8), 5-fluorouracil treated rats (*n* = 8) and combination treated rats from the 70th to the 98th day during the experiment

	70th day			98th day		
	Aidi	5-fluorouracil	Combination	Aidi	5-fluorouracil	Combination
1,3-diaminopropane	682.2 ± 407.5*	147.9 ± 47.4*	250.3 ± 97.4*	551.5 ± 296.8*	798 ± 138*	240.4 ± 88.7*
putrescine	4125 ± 1616	3893 ± 395	3379 ± 1651	4300 ± 1263	3729 ± 953	2613 ± 1490
cadaverine	836 ± 158*	665.2 ± 281.9*	399.2 ± 194.2*	839 ± 424*	1791 ± 353*	323.6 ± 158.4*
spermine	3.776 × 10^4^ ± 2.130 × 10^4^	4.862 × 10^4^ ± 1.554 × 10^4^*	4.515 × 10^4^ ± 2.034 × 10^4^*	3.064 × 10^4^ ± 2.138 × 10^4^	1.500 × 10^4^ ± 0.319 × 10^4^	4.163 × 10^4^ ± 1.606 × 10^4^
spermidine	9.03 × 10^3^ ± 2.79 × 10^3^	1.137 × 10^4^ ± 0.127 × 10^4^	8.21 × 10^3^ ± 3.43 × 10^3^	7719 ± 1231	9.25 × 10^3^ ± 0.60 × 10^3^	7134 ± 2148
N-acetylputrescine	1.83 ± 0.83*	2.45 ± 0.74*	4.93 ± 1.62	1.68 ± 1.84	1.46 ± 0.29*	1.33 ± 0.65*
N-acetylspermine	1.60 ± 0.73*	2.22 ± 0.55	1.94 ± 0.60	1.70 ± 0.57	1.65 ± 0.29*	1.71 ± 0.79
N-acetylspermidine	1.57 ± 0.45*	2.92 ± 0.52	2.08 ± 0.59	1.77 ± 0.40	2.07 ± 1.98	2.26 ± 0.50
γ-aminobutyric acid	2282 ± 508	4219 ± 1312	2116 ± 637	2199 ± 552	1854 ± 277	1960 ± 629
agmatine	46.23 ± 19.67	55.38 ± 18.56	46.09 ± 11.08	33.27 ± 12.41	41.41 ± 10.68	44.69 ± 4.12
L-arginine	2.537 × 10^4^ ± 0.654 × 10^4^	1.691 × 10^4^ ± 0.625 × 10^4^	1.937 × 10^4^ ± 0.233 × 10^4^	2.484 × 10^4^ ± 0.549 × 10^4^	9.98 × 10^3^ ± 3.54 × 10^3^	1.949 × 10^4^ ± 0.161 × 10^4^
lysine	2.967 × 10^4^ ± 0.899 × 10^4^	4.833 × 10^4^ ± 2.406 × 10^4^	2.263 × 10^4^ ± 0.645 × 10^4^*	2.708 × 10^4^ ± 0.804 × 10^4^	2.078 × 10^4^ ± 0.439 × 10^4^	2.132 × 10^4^ ± 0.392 × 10^4^
L-ornithine	6.298 × 10^4^ ± 4.663 × 10^4^	5.968 × 10^4^ ± 0.817 × 10^4^	1.047 × 10^5^ ± 0.926 × 10^5^	7.406 × 10^4^ ± 5.766 × 10^4^	1.028 × 10^5^ ± 0.492 × 10^5^*	1.226 × 10^5^ ± 0.549 × 10^5^
S-adenosyl-L-methionine	213.6 ± 104.0	443.8 ± 192.5	191.7 ± 80.1	217.7 ± 156.3	83.3 ± 67.6	156.3 ± 55.1

(mean ± SD). *p < 0.05, compared with SCCL Rats.

The results of the combination of polyamine metabolome and CYFRA21-1 and SCCAg determination showed both traditional Chinese medicine and synthetic drugs can alter the polyamine metabolic profiling and reduce the level of intracellular polyamines to inhibit proliferation in tumor cells. Also, the phenomenon of altered polyamine levels and polyamine metabolic pathways both confirmed the advantage of Aidi-injection, in which the compound traditional Chinese medicine composed of Cantharides, Ginseng, astragalus and Acanthopanax had the effect of suppressing tumor growth through inhibition of angiogenesis and regulating and strengtheningcellular immune function, with a multi-target and multi-coordinated effect on cancer treatment when combined with synthetic drugs.

In conclusion, both cadaverine and 1,3-diaminopropane have similar characteristics: an increased level in accordance with cancer progression and a gradual decrease according to the treatment until it becomes equal to that in normal rats. Thus, we concluded that cadaverine and 1,3-diaminopropane could serve as potential target biomarkers for monitoring medical treatments and predicting cancer remission.

## DISCUSSION

### Identification of polyamine biomarkers

For further screening of the markers of SCCL, the differences in polyamine metabolomes among healthy, SCCL and treated rats were evaluated using a supervised multivariate analysis. Partial least-squares discriminant analysis (PLS-DA) was performed using SIMCA 13.0. The corresponding variable importance in the projection (VIP values) were also calculated using the PLS-DA model.

**Figure 3:**
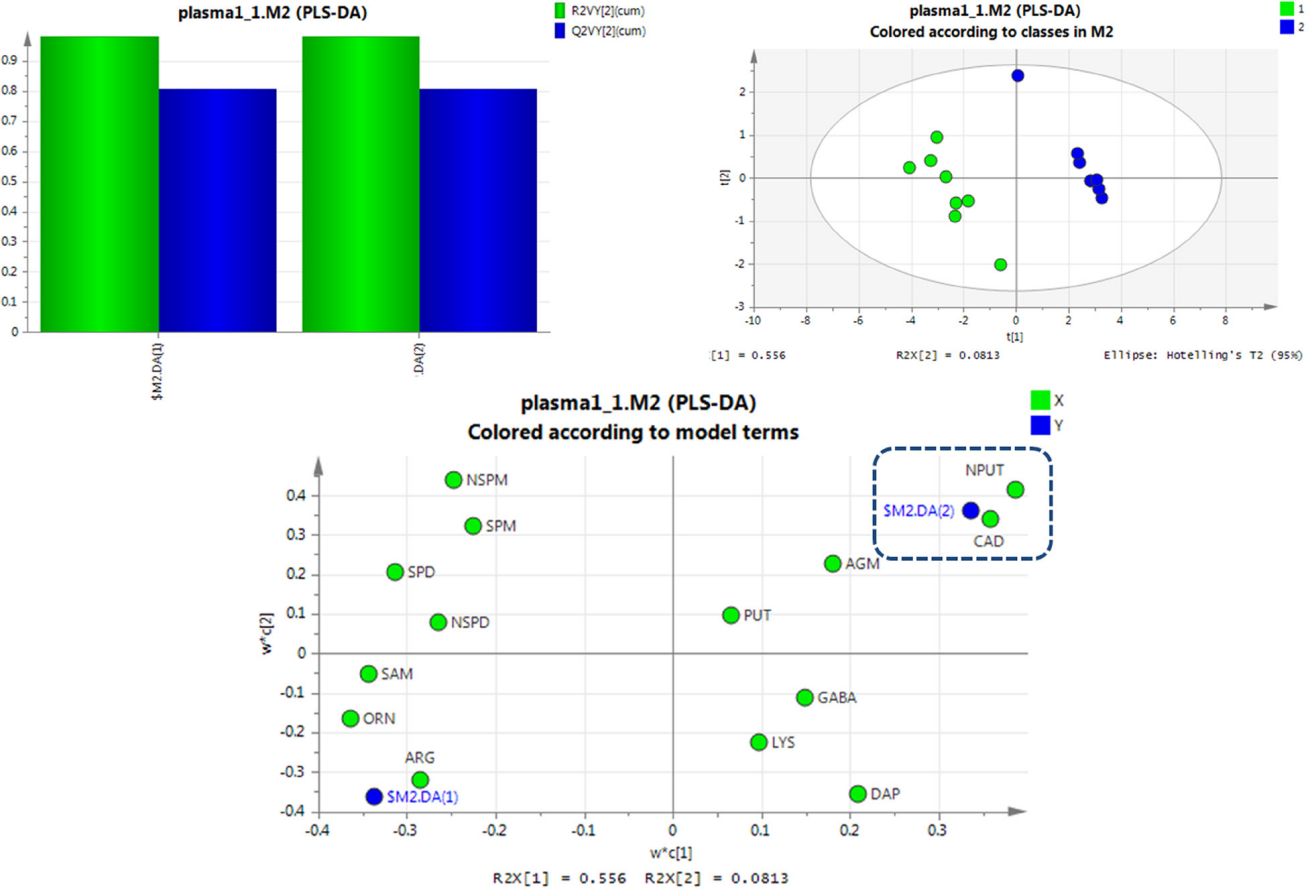
PLS-DA analysis of healthy rats and SCCL rats suffered suffered carcinogenesis for 28 days using polyamines as indicators (DAP: 1,3-diaminopropane, PUT: putrescine, CAD: cadaverine, SPM: spermine, SPD: spermidine, NPUT: N-acetylputrescine, NSPM: N-acetylspermine, NSPD: N-acetylspermidine, GABA: γ-aminobutyric acid, AGM: agmatine, ARG: L-arginine, LYS: lysine, ORN: L-ornithine, SAM: S-adenosyl-L-methionine).

**Figure 4:**
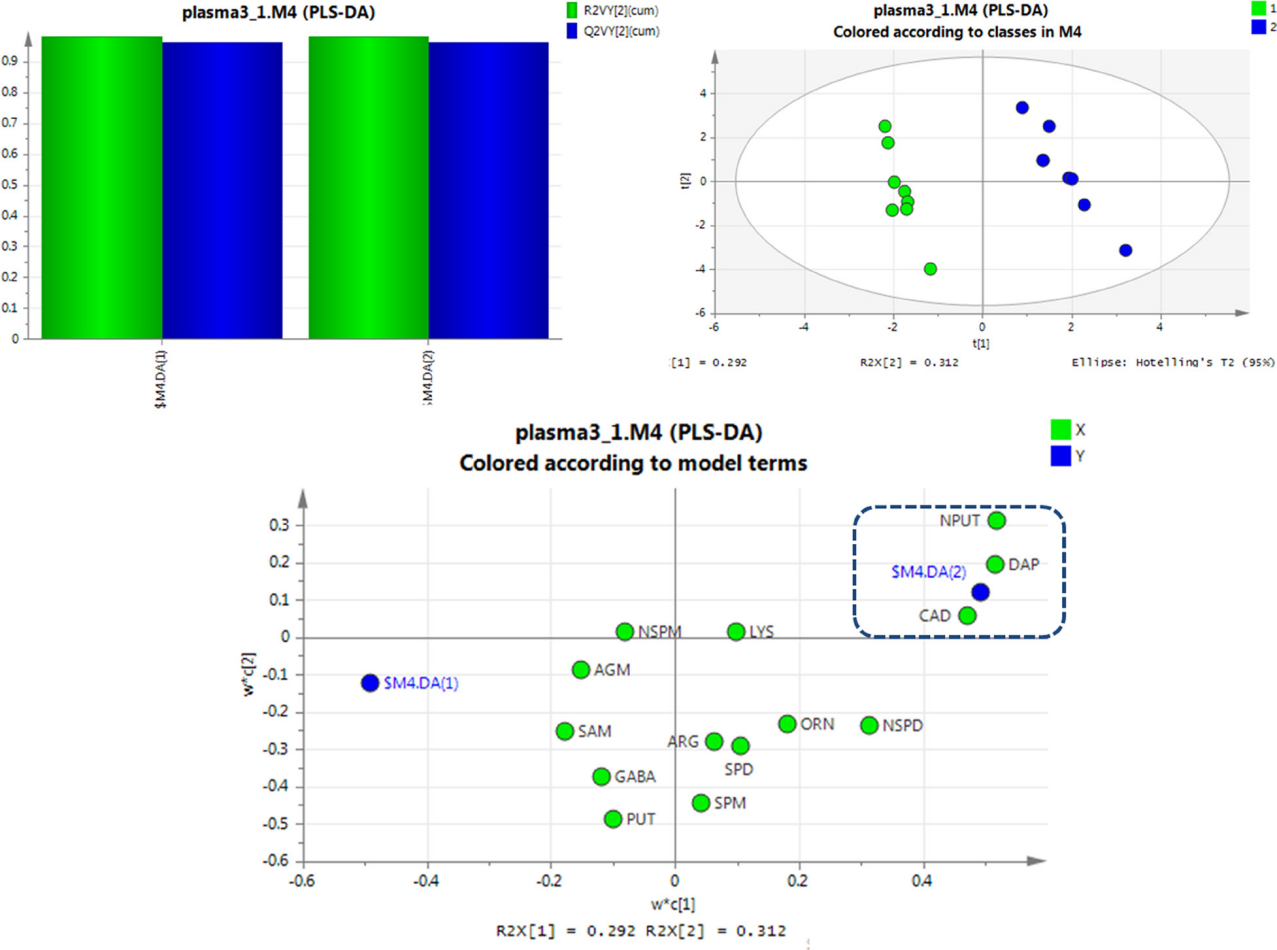
PLS-DA analysis of healthy rats and SCCL rats suffered suffered carcinogenesis for 70 days using polyamines as indicators (DAP: 1,3-diaminopropane, PUT: putrescine, CAD: cadaverine, SPM: spermine, SPD: spermidine, NPUT: N-acetylputrescine, NSPM: N-acetylspermine, NSPD: N-acetylspermidine, GABA: γ-aminobutyric acid, AGM: agmatine, ARG: L-arginine, LYS: lysine, ORN: L-ornithine, SAM: S-adenosyl-L-methionine).

PLS-DA was used to examine the targeted metabolic changes between the SCCL and healthy groups, and the SCCL and treated groups. As shown in Figure 3, the PLS-DA score plot reveals a clear separation between healthy controls and SCCL subjects suffered carcinogenesis for 28 days, with good fitting and predictive performances (R2Y = 0.983, Q2 = 0.809), when using polyamines as indicators. Also, the loading scatter plot and coefficient plot shown in Figure 3 strongly support the effectiveness of using N-acetylputrescine and cadaverine to distinguish SCCL and healthy rats, since their M2.CoeffCS values were more marked than the others. With cancer development, the PLS-DA score plot could also distinguish between the healthy controls and SCCL subjects suffered carcinogenesis for 70 days, with N-acetylputrescine, cadaverine and 1,3-diaminopropane as the most marked polyamines (as shown in Figure 4).

**Figure 5:**
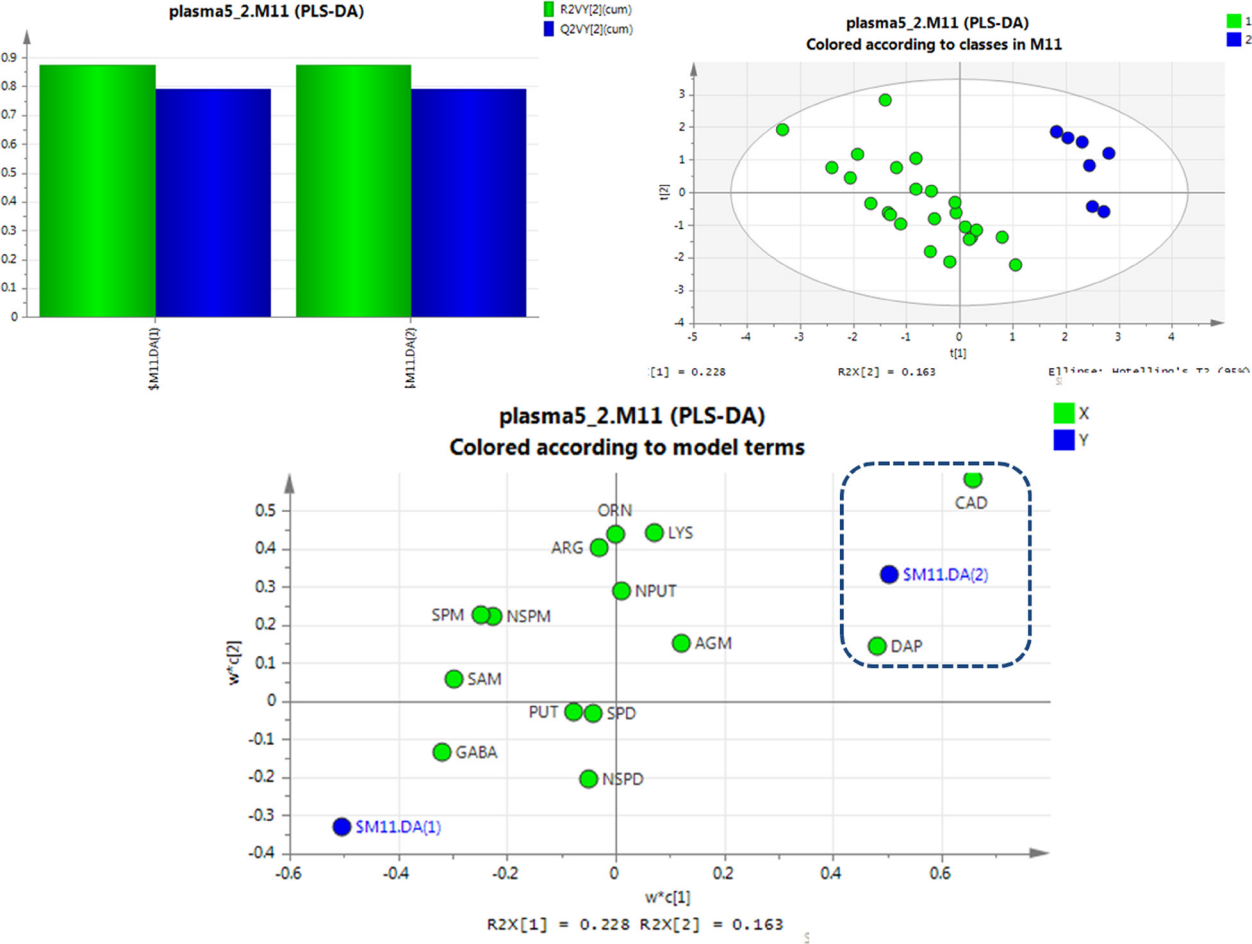
PLS-DA analysis of SCCL rats and treated rats given different treatments for 56 days using polyamines as indicators (DAP: 1,3-diaminopropane, PUT: putrescine, CAD: cadaverine, SPM: spermine, SPD: spermidine, NPUT: N-acetylputrescine, NSPM: N-acetylspermine, NSPD: N-acetylspermidine, GABA: γ-aminobutyric acid, AGM: agmatine, ARG: L-arginine, LYS: lysine, ORN: L-ornithine, SAM: S-adenosyl-L-methionine).

Interestingly, excellent separations were also achieved using PLS-DA analyses for rats given different treatments for 56 days versus SCCL rats (as shown in Figure 5). The results obtained showed that cadaverine and 1,3-diaminopropane can be used as indicators to effectively distinguish between treated and SCCL rats. This agrees with the results of the polyamine metabolic profiles in SCCL and treated rats using the *T*-test. Also, as SCCL treatment progressed, this difference became clearer.

Using the results obtained from the UHPLC-MS/MS and PLS-DA analysis, Receiver Operating Characteristic (ROC) curve analysis was further conducted by SPSS Statistics 18 software (SPSS Inc.) to evaluate the predictive power of each differential polyamine as we did with the target metabolomics on SCCL.

**Figure 6:**
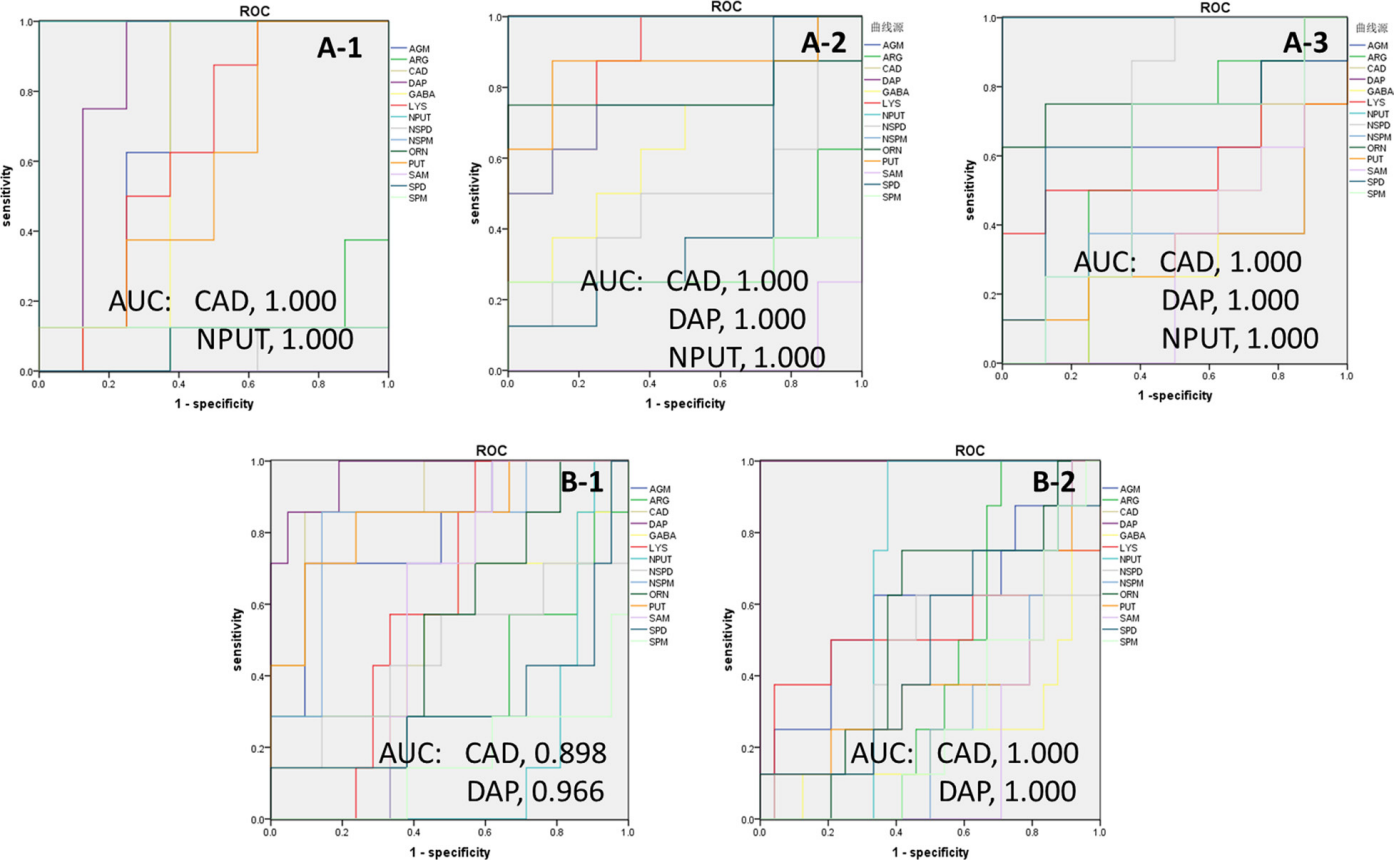
Univariate receiver operating characteristic curve (ROC) analyses for biomarker identification Using a bootstrapping approach to compute the 95% confidence interval (CI) for a single cut-off or for the complete ROC curve. The AUC value of the potential biomarkers for SCCL and treatment are plotted. A-1: SCCL model rats after carcinogenesis for 28 days; A-2: SCCL model rats after carcinogenesis for 70 days; A-3: SCCL model rats after carcinogenesis for 98 days; B-1: SCCL model rats after therapeutic treatment by three kinds of drug for 28 days; B-2: SCCL model rats after therapeutic treatment by three kinds of drug for 56 days; (DAP: 1,3-diaminopropane, PUT: putrescine, CAD: cadaverine, SPM: spermine, SPD: spermidine, NPUT: N-acetylputrescine, NSPM: N-acetylspermine, NSPD: N-acetylspermidine, GABA: γ-aminobutyric acid, AGM: agmatine, ARG: L-arginine, LYS: lysine, ORN: L-ornithine, SAM: S-adenosyl-L-methionine). (Supplementary Tables 1 and 2 listed the detailed results of ROC analysis).

ROC curves were plotted on the basis of the set of optimal sensitivity and specificity values of a diagnostic test at different cutpoints. The cutpoint was determined for each potential biomarker by searching for those that yielded both high sensitivity and specificity. The area under the curve (AUC) was calculated by numerical integration of the ROC curves. The metabolite signature which had the largest area under the ROC curve was identified as having the strongest predictive power for detecting SCCL. The significance threshold of the area under the ROC curve was set at 0.85. The detailed statistics of the area under the ROC curves (AUC), and the corresponding sensitivities and specificities for each of the potential biomarkers for SCCL prediction are listed in Figure 6.

From the results, N-acetylputrescine and 1,3-diaminopropane could be used as early biomarkers of SCCL, and cadaverine could be used for the development of biomarkers of SCCL. Moreover, cadaverine and 1,3-diaminopropane could be used as biomarkers for evaluating the therapeutic effect of anti-cancer drugs on SCCL.

Acetylation reactions may provide a way for cells to reduce the interactions of polyamines with different negatively charged compounds, such as DNA, and RNA, by reducing the number of positive charges they bear. Reduced excretion of N-acetylputrescine may be one of the mechanisms by which the intracellular concentration of polyamines is uncontrolled, and this follows the development of SCCL. Also, 1,3-diaminopropane, derived from S-adenosy-L-methionine by S-adenosylmethionine decarboxylase, is involved in the formation of spermidine and spermine. The increased level of 1,3-diaminopropane in the development of SCCL and its reduced level in the treatment of SCCL can be interpreted as showing that the interactions of spermidine and spermine with DNA analogues can be changed during the progression of cancer. For the same reason, cadaverine, derived from lysine, could serve as a cancer indicator during the treatment stage of SCCL.

## MATERIALS AND METHODS

### Materials and treatments

The reference standards of 1,3-diaminopropane, putrescine, cadaverine hydrochloride, spermidine hydrochloride, spermine, agmatine sulfate salt, N-acetylputrescine hydrochloride, N-acetylspermine trihydrochloride, N-acetylspermidine dihydrochloride, L-ornithine hydrochloride, lysine, L-arginine, S-adenosyl-L-methionine, γ-aminobutyric acid and 1, 6-diaminohexane (used as an internal standard) were all obtained from Sigma-Aldrich (St. Louis, MO). HPLC grade methanol was purchased from Fisher Chemicals (Fair Lawn, NJ). Heptafluorobutyric acid (HFBA) was obtained from Sigma-Aldrich (St. Louis, MO) and all the other reagents were of analytic grade. Redistilled and deionized water was used throughout the study.

3-Methylcholanthrene and diethylnitrosamine were obtained from Sigma-Aldrich (St. Louis, MO) and 5-fluorouracilat were purchased from Shanghai Xudong Haipu Pharmaceutical CO., LTD. (Shanghai, China). Aidi injections are an effective Chinese herbal preparations with anti-cancer activity used for the treatment of liver, lung and colorectal cancer, and they have been used clinically for many years [24, 25]. The Aidi injections were prepared according to the Ministerial Standards of Chinese Medicine. They contain Mylabris (the dried polypide of Mylabris phalerata Pall or Mylabris cichorii Linnaeus), Ginseng Radix Et Rhizoma (the dried root and rhizome of Panax ginseng C.A.Mey.), Astragali Radix (the dried root of Astragalus membranaceus (Fisch.) Bge.var.mongholicus (Bge.) Hsiao, and Acanthopanacis Senticosi Radix Et Rhizoma Seu Caulis (the dried root and rhizome or of Acanthopanax senticosus (Rupr.et Maxim.) Harms). All the treatments used in our experiments were prepared immediately prior to use.

The activities of CYFRA21-1 and SCCAg were determined using ELISA (Enzyme Linked Immunosorbent Assay) assay kits according to the manfacturer’s instructions (Supplementary Table 4 listed the detailed results of ELISA assay).

### Experimental animals and sample collection

Ninety-six male and female Wistar rats (200–220 g) were obtained from the Experimental Animal Center of Shenyang Pharmaceutical University and fed standard rodent chow and given unlimited access to water in an air-conditioned animal center at a temperature of 22 ± 2°C and a relative humidity of 50 ± 10%, with a natural light–dark cycle during the experiment period. The animal studies were carried out according to the Guidelines of Animal Experimentation of Shenyang Pharmaceutical University, and the protocol was approved by the Animal Ethics Committee of the institution.

The study was performed after the rats allowed to acclimatize for one week. The rats were first divided into two groups, SCCL model rats (*n* = 72) and normal rats (*n* = 24). After basal anesthesia produced by intraperitoneal injection of 10% chloral hydrate, the SCCL model rats had cancer induced by intratracheal instillation of 3-methylcholanthrene (MCA, 10 mg/0.1 ml), while diethylnitrosamine (DEN, 0.01 mg/0.1 ml) was also given as the oncogenic agent [26–27]. Normal rats received the same volume of vehicle (iodipin) without MCA and DEN at the same time. During the period of carcinogenesis, plasma and lung samples were collected from the SCCL model rats (*n* = 8) and normal rats (*n* = 8) on the 28th day. Then, on the 42th day, the SCCL model rats (*n* = 64) were divided into four groups, an SCCL group (*n* = 16), a 5-fluorouracil treated group (given 5-fluorouracil injections of 20 mg/kg once a week, *n* = 16), an Aidi injection group (given Aidi injections of 4 mL/kg three times a week, *n* = 16), and a combined medication group (given Aidi injections three times a week and 5-fluorouracil injections once a week, *n* = 16). On the 70th and 98th day, twenty-four hours after the last administration, plasma and lung samples were collected from each group (*n* = 8 for each group) and immediately frozen at −80°C until analysis. Histopathological examination on lung samples was used to confirm the success of the SCCL model and to examine the therapeutic effects of the medicines. Each group contained aqual numbers of male and female rats throughout the experiment.

### Polyamine detection and analysis

For determination of the polyamine metabolome, we used a simple and sensitive UHPLC-MS/MS method described in detail in our previous paper [23]. Briefly, chromatographic separation was achieved with gradient elution using a mobile phase composed of 0.05% heptafluorobutyric acid (HFBA) in water (A) and 0.05% HFBA in methanol (B). MS detection was carried out using a QTRAP™ 4000 MS/MS system from Applied AB Sciex equipped with a Turbo Ion Spray source (Foster City, CA, USA). The detection of the analytes was in multiple reaction monitoring mode (MRM) using electrospray positive ionization (ESI^+^). Each 250 μL plasma sample was deproteinization by the addition of 250 μL methanol (containing 0.1% acetic acid), vortex mixed for 5 min and then centrifuged for 3 min at 15,000 rpm and 4°C. The supernatant was transferred to another Eppendorf microtube and then evaporated to dryness at 30°C under a stream of air. The residue was dissolved in 50 μL methanol (containing 0.05% HFBA) –water (containing 0.05% HFBA) (20:80, *v/v*) and 5 μL of the supernatant was injected for analysis. Lung samples were first weighed on an electronic analytical balance from METTLER TOLEDO (Greifensee, Switzerland), and then homogenized in a 10-fold volume of methanol–water (20:80, *v/v*), vortex mixed for 5 min and centrifuged for 3 min at 15,000 rpm and 4°C, and 5 μL of supernatant was injected for analysis.

### Statistical testing

Polyamine concentrations were obtained from calibration curves and expressed as mean ± SD. The results were presented as mean ± SD and analyzed statistically with Student’s *t*-test and the nonparametric Mann–Whitney test using SPSS 19.0 software for Windows (SPSS Inc., Chicago, IL). The threshold of significance was set at *P* < 0.05. PLS-DA and ROC analysis was also carried out using SIMAC-P (Umetrics AB, Umea, Sweden) and SPSS 19.0, respectively.

## CONCLUSIONS

A plasma polyamine metabolic profile analysis was performed using UHPLC-MS/MS detection following intratracheal instillation of 3-methylcholanthrene in rats during the process of SCCL development and treatment, and 14 polyamine metabolome were detected. Combinating PLS-DA and ROC analysis, it had been shown that N-acetylputrescine and 1,3-diaminopropane may be used as biomarkers to diagnose lung cancer at an early stage, as they were significantly increased in SCCL rats compared with healthy rats. Also, during treatment with the three therapeutic options, the levels of cadaverine and 1,3-diaminopropane decreased with anti-cancer therapy until they become equal to the levels in the control rats, and could be used as biomarkers for evaluating the therapeutic effect of anti-cancer drugs on SCCL. These results may explain the metabolic disturbance in SCCL progression and their recovery by the anti-cancer drugs. Furthermore, the present polyamine profiling combined with quantitative targeted metabolomics methodology will be useful for early cancer screening and anti-cancer drug monitoring.

## SUPPLEMENTARY MATERIALS TABLES




