# Supplementary material for: Plasma N-acetylputrescine, cadaverine and 1,3-diaminopropane: potential biomarkers of lung cancer used to evaluate the efficacy of anticancer drugs
Source: Oncotarget. 2017 Jul 17;8(51):88575–85. doi: 10.18632/oncotarget.19304 (PMC5687628; doi:10.18632/oncotarget.19304)
Supplement: Supplementary file 1 [file oncotarget-08-88575-s001.pdf]

Plasma N-acetylputrescine, cadaverine and 1,3-diaminopropane: potential biomarkers of lung cancer used to evaluate the efficacy of anticancer drugs

SUPPLEMENTARY MATERIALS

Supplementary Table 1: Univariate receiver operating characteristic curve (ROC) analyses for SCCL model rats

ROC analysis for SCCL model rats after carcinogenesis for 28 days						ROC analysis for SCCL model rats after carcinogenesis for 70 days						ROC analysis for SCCL model rats after carcinogenesis for 98 days					
variables	AUC	SEa	Sig.b	95 % CI		variables	AUC	SEa	Sig.b	95 % CI		variables	AUC	SEa	Sig.b	95 % CI	
				up	low					up	low					up	low
**AGM**	.734	.140	.115	.460	1.000	**AGM**	.750	.132	.093	.491	1.000	**AGM**	.484	.153	.916	.184	.785
**ARG**	.109	.086	.009	.000	.279	**ARG**	.313	.152	.208	.015	.610	**ARG**	.594	.154	.529	.292	.895
**CAD**	1.000	.000	.001	1.000	1.000	**CAD**	1.000	.000	.001	1.000	1.000	**CAD**	1.000	.000	.001	1.000	1.000
**DAP**	.844	.119	.021	.610	1.000	**DAP**	1.000	.000	.001	1.000	1.000	**DAP**	1.000	.000	.001	1.000	1.000
**GABA**	.656	.161	.294	.340	.972	**GABA**	.641	.142	.345	.362	.919	**GABA**	.344	.141	.294	.067	.621
**LYS**	.641	.148	.345	.350	.931	**LYS**	.875	.088	.012	.703	1.000	**LYS**	.563	.159	.674	.251	.874
**NPUT**	1.000	.000	.001	1.000	1.000	**NPUT**	1.000	.000	.001	1.000	1.000	**NPUT**	1.000	.000	.001	1.000	1.000
**NSPD**	.047	.053	.002	.000	.150	**NSPD**	.484	.153	.916	.184	.785	**NSPD**	.844	.100	.021	.648	1.000
**NSPM**	.125	.117	.012	.000	.354	**NSPM**	.734	.139	.115	.463	1.000	**NSPM**	.375	.153	.401	.076	.674
**ORN**	.000	.000	.001	.000	.000	**ORN**	.781	.138	.059	.510	1.000	**ORN**	.781	.130	.059	.526	1.000
**PUT**	.578	.152	.600	.281	.875	**PUT**	.859	.108	.016	.648	1.000	**PUT**	.328	.145	.248	.045	.612
**SAM**	.000	.000	.001	.000	.000	**SAM**	.031	.039	.002	.000	.108	**SAM**	.297	.136	.172	.030	.564
**SPD**	.078	.079	.005	.000	.232	**SPD**	.438	.156	.674	.132	.743	**SPD**	.672	.146	.248	.386	.958
**SPM**	.125	.117	.012	.000	.354	**SPM**	.281	.152	.141	.000	.579	**SPM**	.563	.153	.674	.262	.863

Using a bootstrapping approach to compute the 95 % confidence interval (CI) for a single cut-off or for the complete ROC curve.

Supplementary Table 2: Univariate receiver operating characteristic curve (ROC) analyses for SCCL model rats after therapeutic treatment

ROC analysis for SCCL model rats after therapeutic treatment by three kinds of drug for 28 days						ROC analysis forSCCL model rats after therapeutic treatment by three kinds of drug for 56 days					
				95% confidence interval						95% confidence interval	
variables	AUC	SEa	Sig.b	up	low	variables	AUC	SEa	Sig.b	up	low
AGM	.803	.100	.018	.607	.998	AGM	.604	.127	.384	.355	.853
ARG	.415	.154	.507	.114	.716	ARG	.411	.096	.459	.223	.600
CAD	.898	.067	.002	.768	1.000	CAD	1.000	.000	.000	1.000	1.000
DAP	.966	.032	.000	.903	1.000	DAP	1.000	.000	.000	1.000	1.000
GABA	.503	.132	.979	.245	.762	GABA	.229	.110	.024	.014	.444
LYS	.605	.104	.411	.402	.809	LYS	.526	.149	.828	.234	.818
NPUT	.170	.077	.010	.019	.321	NPUT	.656	.096	.192	.469	.844
NSPD	.469	.149	.811	.178	.761	NSPD	.432	.135	.572	.168	.697
NSPM	.816	.100	.014	.620	1.000	NSPM	.276	.090	.061	.100	.452
ORN	.476	.109	.853	.262	.690	ORN	.536	.112	.761	.316	.756
PUT	.844	.094	.007	.660	1.000	PUT	.385	.130	.338	.131	.640
SAM	.571	.105	.577	.366	.777	SAM	.203	.077	.013	.053	.354
SPD	.313	.137	.145	.044	.582	SPD	.469	.121	.794	.231	.707
SPM	.156	.094	.007	.000	.341	SPM	.276	.091	.061	.097	.455

Using a bootstrapping approach to compute the 95% confidence interval (CI) for a single cut-off or for the complete ROC curve.

Supplementary Table 3: The calibration validation for the analytes in plasma. See Supplementary_ Table_3

Supplementary Table 4: Amounts of enzymatic activity in plasma (ng/mL) from normal rats (*n* = 8) and SCCL rats (*n* = 8) from 28th to 98th day in the experiment

	28th day		70th day		98th day	
	**Normal Rats**	**SCCL Rats**	**Normal Rats**	**SCCL Rats**	**Normal Rats**	**SCCL Rats**
Ornithine Decarboxylase	1.00 ± 0.27	1.93 ± 0.15*	1.13 ± 0.30	1.93 ± 0.06*	0.98 ± 0.25	1.77 ± 0.49*
Arginine Decarboxylase	2.77 ± 0.36	3.90 ± 0.15*	2.94 ± 0.98	3.31 ± 0.59*	2.79 ± 0.03	3.17 ± 0.89*
S-adenosine Methionine Decarboxylase	7.07 ± 1.50	9.50 ± 1.12*	6.94 ± 0.79	8.76 ± 0.58*	8.34 ± 2.74	6.99 ± 3.22
Arginine Decarboxylase	8.98 ± 3.21	7.82 ± 0.87	7.79 ± 4.67	8.04 ± 0.52	8.26 ± 3.57	7.18 ± 1.23
Ornithine Decarboxylase Antizyme 1	1.88 ± 0.79	3.93 ± 0.80*	2.03 ± 1.00	3.83 ± 1.39*	1.73 ± 0.22	2.48 ± 0.83*
Polyamine Oxidase	1.38 ± 0.07	1.36 ± 0.20	1.30 ± 0.38	1.32 ± 0.11	1.32 ± 0.27	1.47 ± 0.12
Spermidine and Spermine Acetyl Transferase	35.88 ± 4.69	48.65 ± 4.80*	37.32 ± 3.88	47.56 ± 6.64*	38.45 ± 6.33	42.09 ± 6.79*
Spermine Synthase	3.72 ± 2.08	2.85 ± 0.67	3.88 ± 2.89	2.85 ± 0.48	3.93 ± 2.04	2.34 ± 0.31
Diamine Oxidase	13.52 ± 4.33	13.51 ± 3.26	13.09 ± 3.84	9.89 ± 3.76	13.68 ± 4.21	13.16 ± 4.06
Lysine Decarboxylase	4.53 ± 1.17	7.35 ± 0.87*	4.39 ± 1.28	7.06 ± 1.05*	4.82 ± 0.86	6.56 ± 1.24*

(mean ± SD). *$p < 0.05$, compared to Normal Rats.

Amounts of enzymatic activity in plasma (ng/mL) from Aidi injection medication rats (*n* = 8), 5-fluorouracil injection medication rats (*n* = 8) and combination medication rats from 70th to 98th day in the experiment

	70th day			98th day		
	**Aidi**	**5-fluorouracil**	**Combination**	**Aidi**	**5-fluorouracil**	**Combination**
Ornithine Decarboxylase	1.20 ± 0.38	1.26 ± 0.49	1.08 ± 0.53	1.15 ± 0.21	1.17 ± 0.14	1.27 ± 0.45
Arginine Decarboxylase	4.39 ± 0.47*	4.91 ± 1.58*	4.09 ± 0.71*	4.60 ± 0.38*	4.92 ± 1.20*	4.16 ± 0.24*
S-adenosine Methionine Decarboxylase	6.80 ± 0.98*	6.33 ± 1.62*	6.82 ± 2.39*	5.20 ± 0.90*	5.55 ± 0.36*	5.44 ± 3.81*
Arginine Decarboxylase	8.61 ± 3.13	9.59 ± 1.88*	9.21 ± 0.53*	5.68 ± 0.98*	5.78 ± 1.10*	5.88 ± 2.84*
Ornithine Decarboxylase Antizyme 1	4.60 ± 0.15*	4.23 ± 0.18*	4.41 ± 0.85*	3.49 ± 0.28*	3.42 ± 0.52*	3.84 ± 0.15*
Polyamine Oxidase	1.45 ± 0.26	1.63 ± 0.28	1.51 ± 0.27	1.35 ± 0.11	1.57 ± 0.20	1.48 ± 0.15
Spermidine and Spermine Acetyl Transferase	26.88 ± 7.31*	58.13 ± 2.06*	54.28 ± 1.92*	49.20 ± 5.66*	39.28 ± 5.30	49.13 ± 0.07*
Spermine Synthase	1.90 ± 0.49*	3.21 ± 0.64	3.59 ± 0.24	2.89 ± 0.59	3.96 ± 1.34	3.20 ± 0.48
Diamine Oxidase	13.12 ± 2.76	20.34 ± 3.03	7.73 ± 1.63*	12.91 ± 4.35	8.25 ± 0.16*	14.93 ± 3.10
Lysine Decarboxylase	3.01 ± 0.60*	3.62 ± 0.99*	3.67 ± 0.45*	3.12 ± 0.47*	3.21 ± 0.33*	3.27 ± 0.77*

(mean ± SD). *$p < 0.05$, compared to SCCL Rats.
